# Late Effects of Chronic Low Dose Rate Total Body Irradiation on the Heart Proteome of ApoE^−/−^ Mice Resemble Premature Cardiac Ageing

**DOI:** 10.3390/cancers15133417

**Published:** 2023-06-29

**Authors:** Omid Azimzadeh, Juliane Merl-Pham, Vikram Subramanian, Kateryna Oleksenko, Franziska Krumm, Mariateresa Mancuso, Emanuela Pasquali, Ignacia B. Tanaka, Satoshi Tanaka, Michael J. Atkinson, Soile Tapio, Simone Moertl

**Affiliations:** 1 Section of Radiation Biology, Federal Office of Radiation Protection (BfS), 85764 Nauenberg, Germany; franziska.krumm@outlook.de (F.K.); smoertl@bfs.de (S.M.); 2 Metabolomics and Proteomics Core, Helmholtz Zentrum München, German Research Center for Environmental Health GmbH, 85764 Munich, Germany; juliane.merlpham@helmholtz-munich.de; 3 Abboud Cardiovascular Research Center, Division of Cardiovascular Medicine, Department of Internal Medicine, Carver College of Medicine, University of Iowa, Iowa City, IA 52242, USA; vikram-subramanian@uiowa.edu; 4 Institute of Radiation Biology, Helmholtz Zentrum München, German Research Center for Environmental Health GmbH, 85764 Neuherberg, Germany; katza.zolo@gmail.com (K.O.); m.j.atkinson@tum.de (M.J.A.); soile.tapio@web.de (S.T.); 5 Laboratory of Biomedical Technologies, Agenzia Nazionale per le Nuove Tecnologie, l’Energia e lo Sviluppo Economico Sostenibile (ENEA), 00196 Rome, Italy; mariateresa.mancuso@enea.it (M.M.); emanuela.pasquali@enea.it (E.P.); 6 Institute for Environmental Sciences (IES), Rokkasho, Aomori 039-3212, Japantanakas@ies.or.jp (S.T.); 7 Radiation Oncology, Klinikum rechts der Isar, Technical University, 80333 Munich, Germany

**Keywords:** ionising radiation, TBI, chronic exposure, cardiovascular disease, ageing, proteomics, PPARα, TGFβ, SIRT, AMPK, heart, oxidative stress

## Abstract

**Simple Summary:**

Epidemiological evidence supports an increased risk of cardiovascular diseases (CVD) at doses and dose rates much lower than previously believed. However, the molecular mechanism involved in cardiac damage after chronic irradiation has not been fully elucidated. Here, we studied the proteome of mice hearts chronically irradiated with a very low dose (1 mGy/day) or with a low dose (20 mGy/day) for 300 days. Changes in the proteome were further validated by immunoblotting, enzyme activity assays, immunohistochemistry, and targeted transcriptomics. The data presented here demonstrate that chronic low-dose total body irradiation induces biological changes in mouse cardiac tissue that resemble the effects of premature cardiac ageing. A comprehensive understanding of the molecular mechanisms of CVD following chronic low dose irradiation will enable the identification of target molecules or pathways which may be used in bioassays for measuring endpoints relevant to radiation risk assessment.

**Abstract:**

Recent epidemiologic studies support an association between chronic low-dose radiation exposure and the development of cardiovascular disease (CVD). The molecular mechanisms underlying the adverse effect of chronic low dose exposure are not fully understood. To address this issue, we have investigated changes in the heart proteome of ApoE deficient (ApoE^−/−^) C57Bl/6 female mice chronically irradiated for 300 days at a very low dose rate (1 mGy/day) or at a low dose rate (20 mGy/day), resulting in cumulative whole-body doses of 0.3 Gy or 6.0 Gy, respectively. The heart proteomes were compared to those of age-matched sham-irradiated ApoE^−/−^ mice using label-free quantitative proteomics. Radiation-induced proteome changes were further validated using immunoblotting, enzyme activity assays, immunohistochemistry or targeted transcriptomics. The analyses showed persistent alterations in the cardiac proteome at both dose rates; however, the effect was more pronounced following higher dose rates. The altered proteins were involved in cardiac energy metabolism, ECM remodelling, oxidative stress, and ageing signalling pathways. The changes in PPARα, SIRT, AMPK, and mTOR signalling pathways were found at both dose rates and in a dose-dependent manner, whereas more changes in glycolysis and ECM remodelling were detected at the lower dose rate. These data provide strong evidence for the possible risk of cardiac injury following chronic low dose irradiation and show that several affected pathways following chronic irradiation overlap with those of ageing-associated heart pathology.

## 1. Introduction

Epidemiologic studies have demonstrated an association between exposure of the heart to doses of ionizing radiation as low as 0.5 Gy and an increased risk of cardiovascular disease (CVD) [1,2]. However, there is emerging epidemiological evidence for an increased risk of CVD at doses and dose rates much lower than previously thought [3,4,5,6]. The association between CVD and chronic low-dose exposure has been further corroborated by a new comprehensive meta-analysis performed on more recent epidemiological data [7]. The analysis suggested a higher relative risk per dose unit at low doses and low dose rates [7,8]. However, unlike exposures to high doses, little is known about the biological mechanisms of CVD caused by chronic low doses and low dose rates, such as occupational exposure.

The long-term (300 days) chronic exposure of the ApoE^−/−^ mice to low dose rates (1 mGy/day; 20 mGy/day) has previously been shown to affect the formation of atherosclerotic plaques and plaque size [9]. The analysis of the cardiac mitochondrial proteome and acetylome in the similarly treated ApoE^−/−^ mice showed that the changes in acetylation patterns following irradiation were associated with a series of alterations in the age-associated heart pathology such as mitochondrial dysfunction, alterations in the metabolic and sirtuin (SIRT) pathway, and reduced oxidative stress response [10]. Importantly, similar findings were observed in the proteomic analysis of formalin-fixed, paraffin-embedded (FFPE) left ventricle samples from Mayak nuclear workers chronically exposed to doses >500 mGy. The analysis of the FFPE heart tissues showed significant changes in the expression of proteins involved in metabolism, oxidative stress, and SIRT pathways in chronically irradiated hearts [11]. Since the study analysed FFPE material from Mayak workers who were on average younger than the controls (non-Mayak workers living in the same locale), it was suggested that the alterations in the proteome were contributing to premature ageing following irradiation [11]. Although human FFPE cardiac tissue offers the best source for information about the molecular mechanisms involved in different heart pathologies, the scarcity of cardiac samples from exposed individuals makes validation through independent methods such as enzyme activity assays or immunoblotting very challenging. Moreover, causality interpretations from human heart autopsies are complicated because of confounding factors such as lifestyle, age, and gender, as well as the lack of longitudinal dosimetry. In contrast, a controlled experimental exposure using age- and sex-matched mice allows the interrogation of effects due to radiation and minimises confounding factors. The goal of the present study was to understand the potential biological mechanisms involved in radiation-induced CVD after low dose rate chronic exposures. For this purpose, we have used ApoE^−/−^ female mice, a well-established and widely used model of human CVD [9,10,12,13,14]. Female mice were selected based on the findings of the lifespan study [15,16] where the female mice exposed to 21 mGy/d showed shorter lifespans (attributed to premature ageing) and significantly increased body weights (predisposed to obesity with no corresponding increase in feed consumption) than the non-irradiated mice [15,16]. The increased body weight of female mice has been also observed in lifetime study after acute low dose exposure [17].

In addition, continuously irradiated (20 mGy/day) female mice in lifespan study showed a significantly elevated lipid content in the liver and sera, as well as increased serum levels of total cholesterol, HDL, glucose, insulin, and adipokines (leptin) [18]. The alterations in the serum lipid profile were known as a predictive index of heart disease [19].

We analysed the alterations in the cardiac proteome of ApoE^−/−^ female mice 300 days after continuous exposure to cumulative doses of 0.3 Gy or 6.0 Gy given either at a very low dose rate (1 mGy/day) or at a low dose rate (20 mGy/day) using mass spectrometry-based quantitative proteomics. The analysis documented alterations in the cardiac proteome following chronic total body irradiation at both dose rates. The altered proteins were associated with changes in metabolism, fibrosis, oxidative stress, and ageing. The analysis showed that the late effects of chronic low dose rate total body irradiation on the heart proteome of ApoE^−/−^ mice resemble premature cardiac ageing. Data presented here provide a better understanding of the molecular mechanisms underlying late cardiac effects following low-dose irradiation. The present study suggests that radiation-accelerated ageing may be a key pathological process contributing to the heart disease observed in individuals chronically exposed to low dose rate radiation.

## 2. Materials and Methods

### 2.1. Animals and Irradiation

Female ApoE^−/−^ mice on the C57BL/6J background (Charles River Laboratories, Calco, Italy) were bred and maintained at the Institute for Environmental Sciences (IES) animal facility under specific pathogen-free (SPF) conditions. All experiments were conducted according to the legal regulations in Japan and the Guidelines for Proper Conduct of Animal Experiments (2006, Science Council of Japan, Cabinet Office, 7-22-34, Roppongi, Minato-ku, Tokyo 106-8555, Japan) (http://www.scj.go.jp/ja/info/kohyo/pdf/kohyo-20-k16-2e.pdf) (accessed on 1 February 2023). The experimental protocols (processing numbers 24-20, 24-21, and 25-16) were reviewed and approved by the Institutional Animal Care and Use Committee (IACUC) of the IES according to the science-based guidelines for Laboratory Animal Care (https://www.ncbi.nlm.nih.gov/books/NBK25422/) (accessed on 1 February 2023). (Approval data: 24-20—PROCARDIO—21 March 2012; 24-21—Acquisition and Breeding of B6.129P2-^Apoetm1/Unc^/J—21 March 2012; 25-11—PROCARDIO-continuation/extension 15 March 2013).

The details of the housing of the animals were described before [15,20]. Mice were housed in plastic cages (floor area 338 cm^2^, Tokiwa Kagaku Kikai Co., 5-11-1 Taito-ku, Ueno, Japan) in groups of four animals. After the random assignment of mice to a dose group, the irradiated groups were moved to the irradiation room and stayed there for the whole period of irradiation (300 days), while the non-irradiated control groups were moved to normal animal rooms [15]. Each irradiation room contained nine racks positioned around the gamma radiation sources, and all cages were placed on the racks with the longest side facing the gamma radiation sources. To maintain an equal average radiation exposure, the cages were rotated clockwise to the next position once per week [15]. The details of the dosimetry have been described before [20]. Briefly, dosimetry was performed using two ionization chambers (1200 mL and 12 mL) with thermoluminescent dosimeters (TLDs). Mouse cage racks were positioned using ionization chambers to ensure dose rates met expectations. Absorbed doses were measured by the insertion of TLDs into the abdominal cavities of mice. The mice were irradiated for 3 days and subsequently killed. The TLDs responses were calibrated using the ionization chamber [20].

Mice were fed with standard rodent chow ad libitum and maintained in 12 h light and dark periods. At 8 weeks of age, total body (TBI) irradiation with gamma rays was applied for 300 days at dose rates of 1 mGy/day or 20 mGy/day using a 137-Cs source (22 h/day), reaching cumulative total doses of 0.3 or 6.0 Gy. Control mice were maintained under the same housing conditions. Mice were euthanized after radiation exposure using CO_2_ asphyxiation. All the animals remained apparently healthy during the conduct of the entire study. Routine observations indicated that all animals appeared healthy during the study. Neoplasms were not detected in any of the animals included in this study. Moreover, there were no significant differences in body weight, heart weight, and heart/body weight ratio between the irradiated mice and the non-irradiated mice.

### 2.2. Protein Extraction and Quantification

Fresh-frozen hearts were ground to a fine powder with a cold (−20 °C) mortar and pestle before being suspended in RIPA lysis buffer. Protein concentrations were determined by the Bradford assay following the manufacturer’s instructions (Thermo Fisher, Waltham, MA, USA). For each group, five mice were used. Protein lysates (10 µg) were digested using a modified filter-aided sample preparation (FASP) protocol [21]. Each resulting sample was analysed separately on an LTQ OrbitrapXL (Thermo Fisher Scientific, Waltham, MA, USA) coupled to an Ultimate 3000 nano-HPLC (Dionex) as described previously [22]. The raw files of the individual measurements were loaded into the Progenesis QI software and analysed as described previously [23,24]. Protein identification was performed using the Mascot search engine (Matrix Science, version 2.5.0) with the Ensembl mouse database. The following search parameters were used: 10 ppm peptide mass tolerance, 0.6 Da fragment mass tolerance, and one missed cleavage. Carbamidomethylation (C) was set as fixed modification, and oxidation (M) and deamidation (N, Q) were allowed as variable modifications. Search results were filtered for a Mascot Percolator score <15 in order to reach a false discovery rate (FDR) of 1%. Search results were reimported into the Progenesis QI software and the resulting summed normalised abundances of unique peptides for every single protein were used for the calculation of abundance ratios and statistical analysis (Student’s *t*-test). For final quantifications, proteins identified with only unique peptides (FDR of 1%) were considered. Proteins with ratios of irradiated to controls greater than 1.30-fold or less than 0.77-fold (*t*-test; *p* < 0.05) were defined as being significantly differentially expressed.

### 2.3. Protein-Protein Interaction and Signalling Network

The significantly differentially expressed proteins were subjected to protein interaction and function analysis. The pathway enrichment analyses were performed using the toolset of g:Profiler (https://biit.cs.ut.ee/gprofiler/) (accessed on 1 February 2023) [25] and Ingenuity^®^ Pathway Analysis (https://www.qiagenbioinformatics.com/products/ingenuity-pathway-analysis) (accessed on 1 February 2023) [25].

### 2.4. Immunoblot Analysis

Immunoblot analysis of the heart protein lysates was performed using anti-PPARα (bs-3614R; ThermoFisher, Wilmington, MA, USA), anti-phospho-PPARα (Ser12) (ab3484; Abcam; Boston, MA, USA), anti-Acot1 (sc-373919; Santa Cruz Biotechnology; Dallas, TX, USA), anti-FASN (#3189, Cell Signaling Technology, Danvers, MA, USA), anti-SIRT1 (#9475S; Cell Signaling Technology, Danvers, MA, USA), anti-SIRT3 (#5490; Cell Signaling Technology, MA, USA), anti-AMPK (#2603, Cell Signaling Technology, Danvers, MA, USA), anti-phospho-AMPK (Thr172) (#2535, Cell Signaling Technology, Danvers, MA, USA), anti-Akt (#9272, Cell Signaling Technology, Danvers, MA, USA), anti-phospho Akt (Ser473) (#4058, Cell Signaling Technology, Danvers, MA, USA), and anti-Glutathione (MAB5310; Merck; Darmstadt, Germany). Anti-Voltage-dependent anion channels antibody (VDAC) (#4661, Cell Signaling Technology, Danvers, MA, USA) was used as the loading control. The blots were incubated with the appropriate horseradish peroxidase-conjugated or alkaline phosphatase-conjugated anti-mouse, anti-rabbit, or anti-goat secondary antibody (Santa Cruz Biotechnology, Dallas, TX, USA) for 2 h at room temperature and developed using the ECL system (GE Healthcare Chicago, Il, USA) or 1-step^TM^ NBT/BCIP method (ThermoFisher, Waltham, MA, USA) following manufacturers’ protocols. Quantification of digitised images was done using ImageJ (http://rsbweb.nih.gov/ij/) (accessed on 1 February 2023).

### 2.5. SMAD and Phospho-SMAD Assay

The expression levels of SMAD 2/3 and phospho-SMAD2 (Ser465/467)/phospho-SMAD3 (Ser423/425) were measured using PathScan^®^ Total Smad2/3 and phospho Smad Sandwich ELISA Kit (Cell Signaling Technology; #12000 and #12001; Danvers, MA, USA) in whole heart lysate according to the manufacturer’s instructions.

### 2.6. Total SIRT Activity Assay

The total SIRT activity was measured in whole heart lysate using a fluorometric assay (ab156915; Abcam, Cambridge, UK) according to the manufacturer’s recommendation.

### 2.7. AMPK Assay

The total 5′ AMP-activated protein kinase (AMPK) and phospho-AMPK (Ser487) were measured in whole heart lysate using a colourimetric assay (#PEL-AMPKA-S487-T-1 RayBio; Peachtree Corners, GA, USA) according to the manufacturer’s recommendation.

### 2.8. mTOR Assay

The total mTOR and phospho-mTOR (Ser2448) were measured in whole heart lysate using a colourimetric assay (#PEL-mTOR-S2448-T-1 RayBio; Peachtree Corners, GA, USA) according to the manufacturer’s recommendation.

### 2.9. GSH/GSSG Ratio Detection Assay

The ratio of GSH/GSSG was measured in whole heart lysate using a fluorometric assay (ab138881, Abcam; Boston, MA, USA) according to the manufacturer’s recommendation.

### 2.10. GLRX Activity Assay

Glutaredoxin (GLRX) activity was measured in whole heart lysate using a fluorometric assay (#500239, Cayman Chemical Company, Ann Arb, MI, USA) as recommended by the manufacturer.

### 2.11. GPX Activity Assay (ab102530)

Glutathione Peroxidase (GPX) activity was measured in whole heart lysate using a colorimetric assay (ab102530, Abcam; Boston, MA, USA) as recommended by the manufacturer.

### 2.12. Lipid Peroxidation Assay

Stress-induced protein modification was measured in whole heart lysate using the lipid peroxidation colourimetric assay (ab118970; Abcam; Boston, MA, USA) according to the manufacturer’s instructions.

### 2.13. Pathway-Focused Gene Expression Profiling with qRT-PCR

Total RNA was extracted from hearts using RNeasy Plus Micro Kit following the manufacturer’s protocol (QIAGEN, Hilden, Germany). The quantity and quality of the total RNA isolated from mice hearts were measured with the Nanodrop spectrophotometer (PeqLab Biotechnology; Erlangen, Germany). Mouse RT^2^ Profiler PCR arrays (QIAGEN, Hilden, Germany) were used to profile the expression of 84 genes related to cellular senescence. Single-stranded cDNA was synthesized from 100 ng of total RNA using the SuperArray reaction-ready first-strand cDNA synthesis kit. The cDNAs were mixed with SuperArray RT^2^ Real-time SYBR Green/ROX PCR master mix and real-time PCR was performed following the manufacturer’s instructions. Thermal cycling and fluorescence detection were performed using a StepOne Sequence Detection System (Applied Biosystems, Foster City, CA, USA) according to the manufacturer’s instructions. Data analysis was performed based on the ΔΔCT method with normalization of the raw data to the housekeeping genes, including glucuronidase beta (Gusb), hypoxanthine-guanine phosphoribosyl transferase (Hprt), heat shock protein 90 alpha (Hsp90ab1), and actin B (Actb). The expression of genes related to cellular senescence was compared between the groups using the student’s *t*-test; mRNAs with ratios of irradiated to controls greater than 1.50-fold or less than 0.66-fold (*t*-test; *p* < 0.05) were defined as being significantly differentially expressed.

### 2.14. Histology

Hearts from controls and chronically irradiated (0.3 and 6 Gy) ApoE^−/−^ mice were fixed in 10% buffered formalin and embedded in paraffin before being sectioned (in thickness of 7 μm) and stained with Masson’s trichrome according to a standard protocol (Masson trichrome with aniline blue, Bio-Optica). The collagen deposition was evaluated in the right ventricle (RV) using the HistoQuest 2.0.2.0249 software (TissueGnostics, Vienna, Austria) and expressed as a percentage of the total RV area.

### 2.15. Statistical Analysis

GraphPad Prism 9.0 was used for all statistical analyses. Statistical analysis was performed using one-way ANOVA, followed by Tukey’s multiple comparisons test for the groups. Statistical significance was accepted when the Adjusted *p* value < 0.05. Data are presented in figures as means of biological replicates ±SEM/± SD.

### 2.16. Data Availability

Data have been deposited into the RBstore public database: https://www.storedb.org/store_v3/study.jsp?studyId=1106 (accessed on 1 February 2023).

## 3. Results

### 3.1. Low Dose Chronic Irradiation Altered the Cardiac Proteome of ApoE ^−/−^ Mice

**Figure 1:**
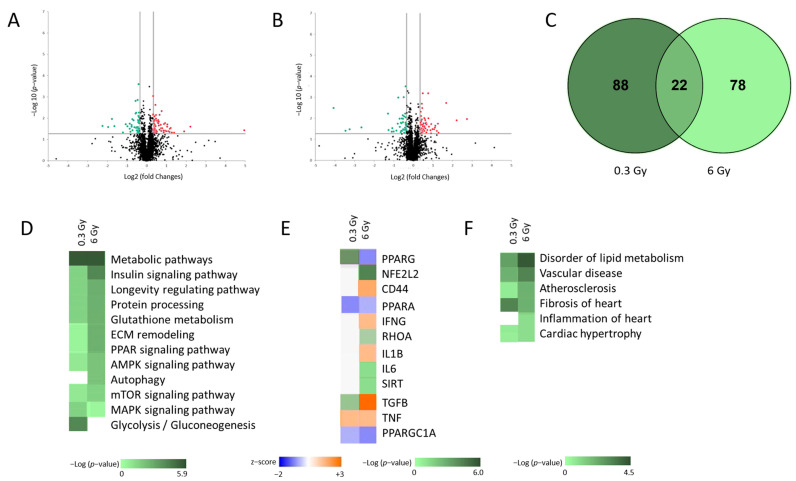
Changes in the proteome of hearts after chronic irradiation. The volcano plot represents the distribution of all quantified proteins in cardiac tissue after chronic exposure to accumulated doses of 0.3 Gy (**A**) and 6 Gy (**B**). Deregulated proteins (±1.3-fold; *p* < 0.05) are highlighted in green (downregulated) and red (upregulated) (**A**,**B**). The full protein names are provided in Table S1. The Venn diagram illustrates the set of significantly deregulated proteins shared between both radiation doses (**C**). The most significant canonical pathways altered in proteome profiles (**D**). The analysis was generated by the toolset of g: Profiler. Graphical representation of different predicted upstream regulators (Supplementary Table S6) (**E**). Graphical representation of predicted cardiac toxicity (**F**). The analyses were performed by Ingenuity^®^ Pathway Analysis and are displayed using a green colour gradient (representative of −log *p*-value), where a darker colour corresponds to a higher statistical significance. A positive z-score implies potential activation (orange) and a negative z-score indicates potential inhibition (blue) of the pathway. The white colour indicates pathways where no prediction can currently be made (Supplementary Table S6).

The changes in the cardiac proteome of chronically irradiated ApoE^−/−^ female mice were analysed with label-free quantitative proteomics. A total of 2531 cardiac proteins were identified and quantified (Supplementary Table S1 and Figure 1A–C). Of these, 110 and 100 proteins were significantly deregulated compared to controls after cumulative radiation doses of 0.3 and 6 Gy, respectively (Figure 1C and Supplementary Tables S2 and S3). The analysis showed no dose-dependent difference in the number of significantly deregulated proteins in irradiated groups. The common 22 proteins (Figure 1C) do not form a functional cluster.

The pathway enrichment analysis was performed using a toolset of g:Profiler (https://biit.cs.ut.ee/gprofiler/) (accessed on 1 February 2023) and Ingenuity Pathway Analysis (IPA) software (https://www.qiagenbio-informatics.com/products/ingenuity-pathway-analysis) (accessed on 1 February 2023). The comparison of the two proteome profiles showed overlapping biological events in irradiated hearts (Supplementary Tables S4 and S5). The comparison of the most affected pathways in the heart after exposure to 0.3 and 6 Gy showed a dose-dependent enrichment (Benjamini-Hochberg FDR approach; *p* < 0.05) of the signalling pathways, such as the metabolic pathway, insulin signalling pathway, longevity regulating pathway, protein processing, glutathione metabolism, ECM-receptor interaction, peroxisome proliferator-activated receptor alpha (PPARα), AMP-activated protein kinase (AMPK), and mammalian target of rapamycin (mTOR) signalling pathways. More proteins identified were involved in the autophagy after 6 Gy while more proteins were enriched in the glycolysis and MAPK after exposure to 0.3 Gy in irradiated heart proteome (Figure 1D and Supplementary Table S6). The analysis also predicted the activation/inhibition of several upstream regulators in irradiated hearts. Tumour necrosis factor alpha (TNFα) was predicted to be induced after irradiation (Figure 1E and Supplementary Table S6) whilst PPARα and peroxisome proliferator-activated receptor gamma coactivator 1 (PGC1) were predicted to be inhibited in irradiated hearts at both doses (Figure 1E and Supplementary Table S6). Transforming growth factor beta (TGFβ), Interleukins 1B (IL1B), interferon-gamma (IFNγ), and CD44 were predicted to be induced and PPARγ inhibited after 6 Gy. Clusters of deregulated proteins were also identified after 6 Gy that belong to the regulatory networks of nuclear factor erythroid 2-related factor 2 (NFE2L2), Ras Homolog Gene Family, Member A (RHOA), IL6, and Sirtuin (SIRT) (Figure 1E and Supplementary Table S6). A number of differentially expressed proteins were associated with different cardiac toxicity, including disorders of lipid metabolism, atherosclerosis, vascular disease, hypertrophy, and fibrosis at both exposures (Figure 1F and Supplementary Table S6). More proteins involved in fibrosis were identified after 0.3 Gy (Figure 1F and Supplementary Table S6). 

### 3.2. Chronic Irradiation Suppresses the Activity of PPARα Signalling Pathway

**Figure 2:**
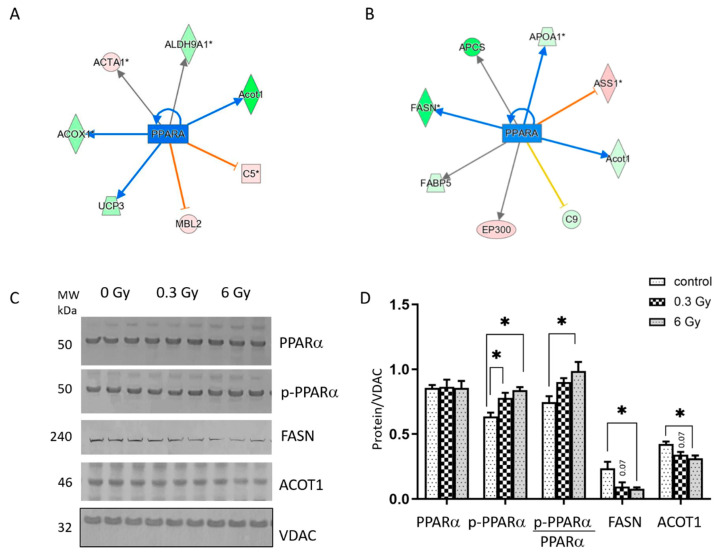
Analysis of the cardiac PPARα and metabolic enzymes after chronic irradiation. Graphical representation of the deregulated protein networks with their upstream transcriptional regulators PPARα after exposure to an accumulated dose of 0.3 Gy (**A**) and 6 Gy (**B**). The upregulated proteins are marked in red and the downregulated are marked in green. The nodes in blue represent transcription factors. A negative z-score indicates potential inhibition (blue) of the transcription factor. The proteins in red are upregulated and in green are downregulated. The analyses were generated using Ingenuity^®^ Pathway Analysis. The full protein names are given in Tables S1–S3. Immunoblot analysis of total and p-PPARα (at Ser12), FASN, and ACOT1 in heart lysate samples is shown (**C**). The columns represent the average ratios of relative protein expression in control and irradiated samples after background correction and normalisation to VDAC expression (**D**) (one-way ANOVA followed by Tukey’s multiple comparisons test; * adjusted *p* value < 0.05; *n* = 3). The error bars represent the standard error of the mean (±SEM).

The metabolic transcription factor PPARα was predicted to be inactivated in irradiated hearts (Figure 1D–F and Figure 2A,B and Supplementary Table S6). This observation was confirmed by immunoblotting showing that the expression of phosphorylated (inactive) PPARα (p-PPARα) was significantly increased in irradiated mice at both doses (Figure 2C,D). The expression level of full-length PPARα remains unchanged. The ratio of p-PPARα to full-length PPARα increased after 6 Gy, suggesting inhibition of the transcription factor activity. The immunoblotting confirmed the decrease in the expression level of the transcriptional targets of PPARα, including Acyl-coenzyme A thioesterase 1 (Acot1) and fatty acid synthase (FASN), following 6 Gy (Figure 2C,D).

### 3.3. Cardiac Oxidative Stress Response Is Affected in Irradiated Hearts

**Figure 3:**
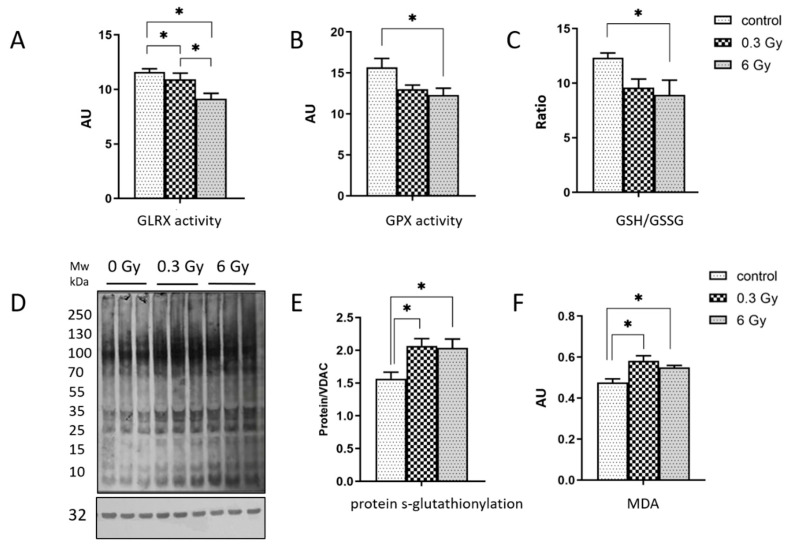
Analysis of the oxidative stress response. The activities of GLXR and GPX were compared in mice hearts (**A**,**B**). The ratio of GSSG/GSH was measured in hearts (**C**). Immunoblot analysis of protein s-glutathionylation is shown (one-way ANOVA followed by Tukey’s multiple comparisons test; * adjusted *p* value < 0.05; *n* = 5) (**D**). The columns represent the average ratios of relative protein s-glutathionylation in the control and irradiated samples after background correction and normalisation to VDAC expression (one-way ANOVA followed by Tukey’s multiple comparisons test; * adjusted *p* value < 0.05; *n* = 3) (**E**). The amount of lipid peroxidation as a marker of oxidative stress was measured in mice hearts using ELISA (one-way ANOVA and Tukey test for multiple comparisons; * adjusted *p* value < 0.05; *n* = 5) (**F**). The error bars represent the standard error of the mean (±SEM).

Based on the proteomics data, the proteins involved in glutathione metabolism including glutathione peroxidase 3 (GPX3) and glutaredoxin (GLRX) were changed in irradiated hearts (Figure 1D,E and Supplementary Table S3), suggesting a marker of alterations in oxidative stress response. To validate the proteomics findings, the activity of the antioxidant enzymes GPX and GLRX, as well as the ratio of GSH/GSSG as a hallmark of oxidative stress and oxidative stress-induced protein modification (lipid peroxidation and protein s-glutathionylation), were compared in heart tissues (Figure 3A–D). The activity of GLRX was decreased in irradiated hearts and in a dose-dependent manner (Figure 3A). The activity of GPX showed a significant reduction in hearts only following exposure to 6 Gy (Figure 3B). The ratio of GSH/GSSG was also reduced in 6Gy irradiated hearts (Figure 3C). The analysis of the amount of protein s-glutathionylation and lipid peroxidation as markers of accumulated oxidative damage showed a significant increase after irradiation (Figure 3D–F).

### 3.4. Chronic Irradiation Affects the Cardiac Fibrogenic Pathway

**Figure 4:**
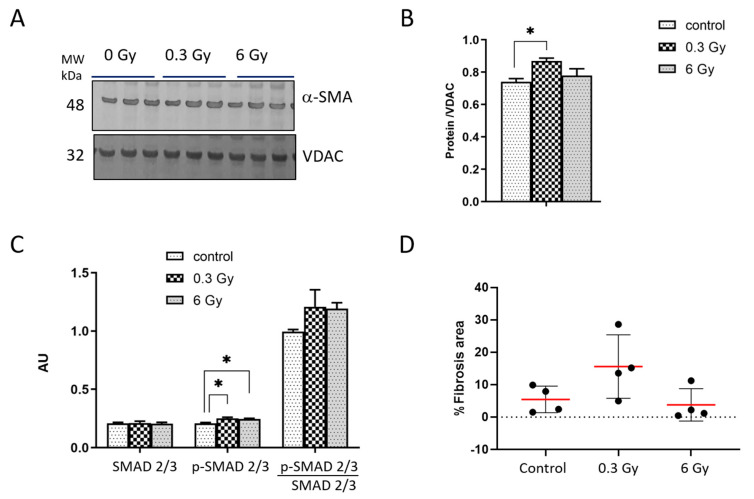
Analysis of the α-SMA and TGF-β signalling pathways. Immunoblot analysis of α-SMA is shown (**A**). The amount of the total protein was measured by Ponceau S staining. The columns represent the average ratios of relative protein expression in control and irradiated samples after background correction and normalisation to VDAC expression (one-way ANOVA followed by Tukey’s multiple comparisons test; * adjusted *p* value < 0.05; *n* = 3) (**B**). The expression levels of SMAD 2/3 and p-SMAD 2/3 were compared in mice hearts using ELISA (one-way ANOVA followed by Tukey’s multiple comparisons test; * adjusted *p* value < 0.05; *n* = 3) (**C**). The error bars represent the standard error of the mean (±SEM). Collagen deposition was evaluated in the RV using the HistoQuest 2.0.2.0249 software (TissueGnostics, Vienna, Austria) and expressed as a percentage of the total RV area (**D**). The red lines indicate the mean and the error bars represent the standard deviations (±SD).

Proteomics analysis showed that several proteins associated with the ECM remodelling and TGFβ pathways were altered in the irradiated heart (Figure 1D–F and Supplementary Table S6). We measured the protein levels of the alpha-smooth muscle actin (α-SMA) as a marker of fibroblast differentiation involved in ECM remodelling and the SMAD family, the key components of canonical TGFα signalling (Figure 4A–C). The analysis showed that α-SMA increased significantly in 0.3 Gy-irradiated hearts. The expression of phosphorylated (active) SMAD2/3 (p-SMAD2/3) was significantly increased in irradiated mice at both doses (Figure 3C).

The increase in the ratio of p-SMAD 2/3 to total SMAD 2/3 levels following irradiation was not statistically significant (Figure 4C). In good agreement with proteomics data, we found an increased level of several collagen isoforms in the proteome of irradiated hearts (Supplementary Tables S2 and S3). To validate this, the deposition of collagen in cardiac tissue was analysed using Masson’s trichrome staining. Representative images are shown (Supplementary Figure S1). On average, the relative area of fibrotic tissue within the RV was 5.44 + 4.1 SD% in the control sections and 15.58 + 9.76 SD% and 3.76 + 5.01 SD% in the heart sections after exposure to 0.3 and 6 Gy, respectively (Figure 4D and Supplementary Figure S1). The fibrotic area was bigger in hearts after exposure to 0.3 Gy compared to the controls and those exposed to 6 Gy; however, this difference did not reach statistical significance.

### 3.5. Expression and Activity of Sirtuins Were Affected in Chronically Irradiated Heart

**Figure 5:**
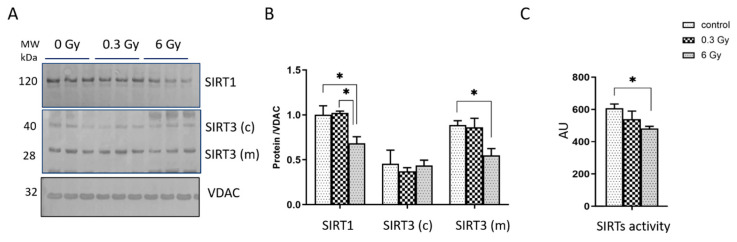
Analysis of the sirtuin proteins activity and expression. Immunoblotting analysis of SIRT1 and SIRT3 was performed in the hearts (**A**,**B**). The columns represent the average ratios of relative protein expression in the control and irradiated samples after background correction and normalisation to VDAC expression (one-way ANOVA and Tukey test for multiple comparisons *t*-test; * adjusted *p* value < 0.05; *n* = 3) (**B**). The activity of total SIRTs was compared between irradiated and control samples (one-way ANOVA followed by Tukey’s multiple comparisons test; * adjusted *p* value < 0.05; *n* = 5) (**C**). The error bars represent the standard error of the mean (±SEM).

To validate the proteomics result indicating an alteration in a cluster of proteins regulated by the sirtuin pathway in irradiated hearts (Figure 1E and Supplementary Table S6), we measured the expression levels of SIRT1 and SIRT3, both mitochondrial (m) and cytoplasmic forms ©, using immunoblotting (Figure 5A,B) and the activity of total SIRT proteins using ELISA in heart tissues (Figure 5C). In good agreement with previous data [10], the analysis showed a decrease in the levels of SIRT1 in 6 Gy-irradiated hearts compared to controls and 0.3 Gy irradiated hearts. The mitochondrial SIRT3 decreased in 6 Gy-irradiated hearts. The cytoplasmic SIRT3 remained unchanged (Figure 5A,B). The activity of total SIRTs was significantly reduced in mouse hearts after 6 Gy irradiation (Figure 5C).

### 3.6. mTOR and AMPK Signalling Pathways were Affected by Chronic Irradiation

**Figure 6:**
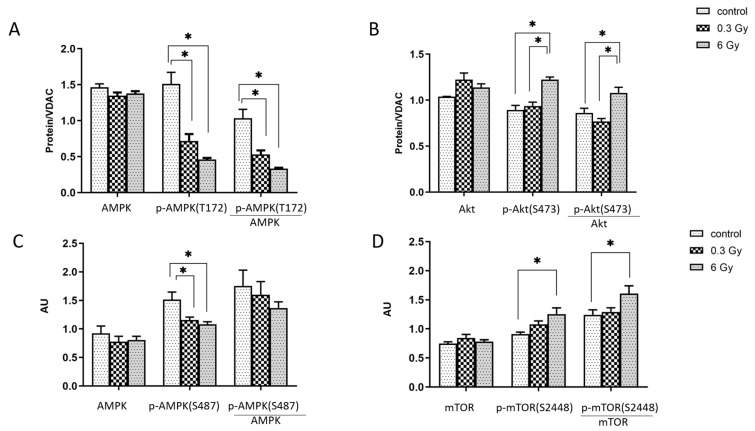
Analysis of the AMPK, Akt, and mTOR. The expression levels of AMPK and phosphorylated -AMPK (T172) (**A**), and Akt and phosphorylated -Akt (**B**) were compared in mice hearts using immunoblotting (one-way ANOVA followed by Tukey’s multiple comparisons test; * adjusted *p* value < 0.05; *n* = 3). The expression levels of AMPK and phosphorylated -AMPK (S487) (**C**), and mTOR and phosphorylated -mTOR (**D**) were compared in mice hearts using ELISA (one-way ANOVA followed by Tukey’s multiple comparisons test; * adjusted *p* value < 0.05; *n* = 5). The error bars represent the standard error of the mean (±SEM).

Proteomics data showed an effect of chronic irradiation on mTOR and AMPK pathways (Figure 1D). Since the activities of the mTOR and AMPK are regulated by phosphorylation, we analysed the levels of phosphorylated and total proteins in mice hearts using immunoblotting (Figure 6A,B) and ELISA (Figure 6,D). To validate the impaired AMPK activity and regulatory role of Akt, we examined the phosphorylation of Thr172 on AMPK and Ser473 of Akt (Figure 6A,B and Supplementary Figure S2). The data showed that the level of p-AMPK (at Thr172) and the ratio of p-AMPK (Thr172)/AMPK decreased in irradiated samples (Figure 6A). Analysis of Akt and p-Akt showed an increase in p-Akt (Ser473) and the ratio of p-Akt/Akt with 6Gy compared to the control and 0.3 Gy-irradiated mice (Figure 6B). Akt has been shown to phosphorylate AMPK at Ser487, resulting in decreased Thr172 phosphorylation [26]. Therefore, we examined the p-AMPK (at Ser487). Unexpectedly, a decrease in the level of p-AMPK (Ser487) was found after irradiation, while the total AMPK level remained unchanged (Figure 6C).

We compared also mTOR activity in hearts. An increase in the level of p-mTOR (at Ser2448) and in the ratio of p-mTOR/mTOR was found in 6 Gy-irradiated hearts while the total mTOR level showed no changes, suggesting a potential activation of the pathway (Figure 6D).

### 3.7. Senescence-Associated Genes Were Altered in Chronically Irradiated Hearts

**Figure 7:**
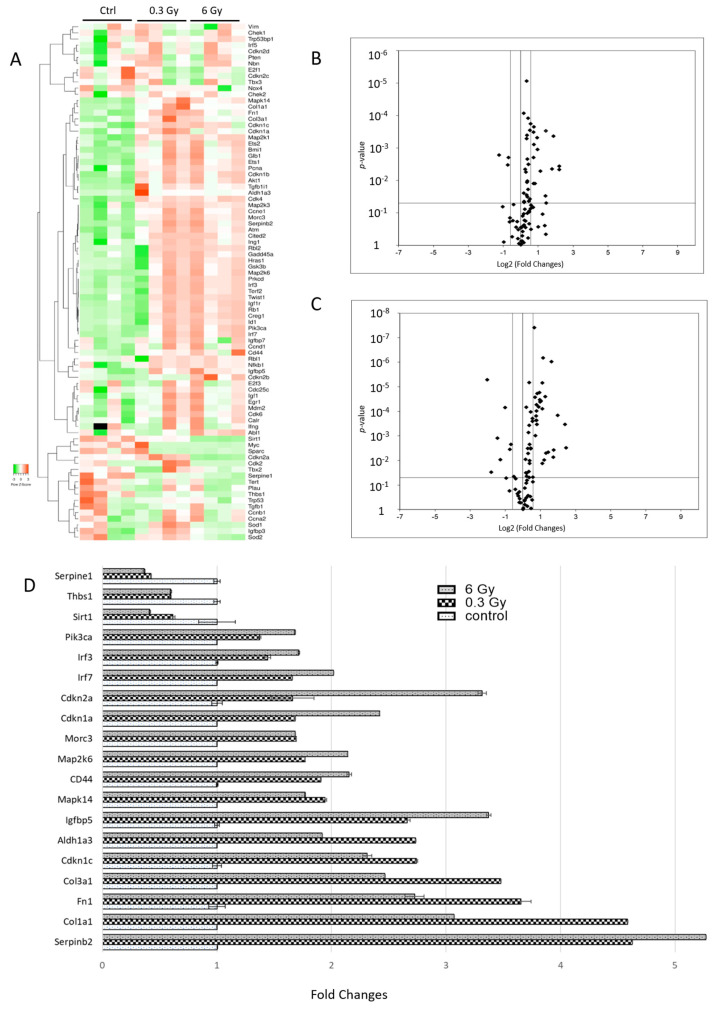
Senescence-associated mRNA expression profiling. The heat map shows hierarchical clustering (average linkage, Spearman ranked correlation) of 84 mRNA relevant to senescence (**A**). The green bars indicate downregulation and the red bars upregulation. The analysis was performed using the Heatmapper web server [27]. The volcano plot represents the distribution of all quantified mRNA in cardiac tissue after chronic exposure to accumulated doses of 0.3 and 6 Gy (**B**,**C**). Data were analysed using the ΔΔCT method with normalization of the raw data to the housekeeping genes. They shared significantly deregulated mRNA in the irradiated hearts (**D**). The error bars represent the standard error of the mean (±SEM) (*t*-test; * *p* < 0.05; *n* = 4).

The observed alterations in several pathways associated with ageing and longevity (including SIRT, AMPK, and mTOR signalling pathways) (Figure 1D,E) prompted us to investigate if radiation exposure is causing an increased level of senescent cells in cardiac tissue. Using the expression of senescence-associated mRNA as a surrogate, we compared the different treatments. Data for individual genes are provided in Table S7. Out of 84 senescence-associated genes examined, 19 and 36 genes showed significant differences in expression between controls and 0.3 and 6 Gy chronically irradiated mice, respectively (Figure 7A–C and Tables S8 and S9). The analysis showed 19 senescence-associated mRNAs are changed by both radiation doses (Tables S8 and S9).

All significantly deregulated mRNAs present in 0.3 Gy-irradiated hearts were also changed at 6 Gy. The levels of several senescence markers such as *CD44*, *Igfbp5*, *Cdkn1a* (*p21*), and *Cdkn2a* (*p16*) were significantly increased in the irradiated samples compared to the controls (Figure 7C), while the *SIRT1*, Plasminogen activator inhibitor 1 (*Serpine1*), and Thrombospondin-1 (*Thbs1*) were decreased after irradiation. The analysis also confirmed the upregulation of fibrosis-related mRNAs including collagen (*Col3a* and *Col1a1*) and fibronectin (*Fn1*), as well as genes involved in the inflammatory response such as Interferon regulatory factors 3 and 7 (*Irf 3* and *Irf 7*) in irradiated hearts (Figure 7C and Tables S8 and S9).

## 4. Discussion

A newly published meta-analysis of updated epidemiological data has emphasized more than before the close association between chronic low-dose radiation exposure and CVD [7]. The analysis showed that low dose radiation and/or low dose rate exposures are associated with an increased risk of CVD per unit dose [8]. However, underlying molecular mechanisms of CVD caused by chronic low dose rate exposure are not fully known.

The analyses showed persistent alterations in the cardiac proteome at both dose rates; however, the effect was more pronounced following a higher dose rate. More importantly, the study shows that chronic radiation exposure of 0.3 Gy delivered at a very low dose rate (1 mGy/day) was sufficient to alter the cardiac proteome, resulting in biologically relevant dysfunction. Several biological processes including energy metabolism, ECM remodelling, longevity pathways, autophagy, and oxidative stress response were affected in the irradiated hearts. The study confirmed changes in several signalling pathways identified previously in the cardiac proteome of the mouse mitochondria and human heart tissues after chronic exposure [10,11,28,29]. In addition to the previously observed changes, we found alterations in pathways that contribute to cardiac longevity and ageing processes, such as AMPK, mTOR, and autophagy signalling pathways [30,31,32,33,34,35,36]. Taken together, these data indicate that the changes in the proteome of the mouse heart induced by chronic low dose irradiation resemble cardiac ageing. Ageing of the heart is a complex process affected by a combination of genetic and environmental factors. Cardiac ageing is associated with a gradual decline in cardiovascular function and accumulation of damage to cardiac tissue. On the functional (biological and mechanistic) level, cardiac ageing is characterized by DNA damage, genomic instability, increased oxidative stress, metabolic and mitochondrial dysfunction, tissue inflammation, and cellular senescence [37,38]. SIRT1, mTOR, and AMPK-signalling pathways are known as regulatory pathways of longevity and ageing of the heart [35,36]. Interplay between the activation of the SIRT/PGC1 pathway and suppression of the Akt/mTOR cascade modulates a broad range of biological processes involved in cellular growth and survival, including metabolism, antioxidative responses, and autophagy [36,39,40]. SIRT and PGC1 can negatively regulate Akt and mTOR, while inhibition of mTOR by rapamycin can induce SIRT. AMPK plays a crucial role to tune the interconnectivity between SIRT1 and Akt/mTOR [36,41]. Interestingly, several signalling pathways contributing to premature ageing are known to be disrupted after radiation exposure [10,42,43,44].

In good agreement with these findings, the proteomics and transcriptomics data presented here showed alterations in several molecules that are involved in the SIRT1, mTOR, and AMPK pathways. Data showed that chronic irradiation decreased the expression and activity of AMPK and SIRT proteins while activating mTOR signalling. We have also previously shown that the SIRT pathway is significantly affected in the human and murine hearts following chronic irradiation [10,43]. The observed decrease in SIRT1 and SIRT3 activity was associated with hyperacetylation of cardiac mitochondrial proteins following chronic irradiation (20 mGy/day for 300 days) in ApoE^−/−^ mice [10]. Enhanced mitochondrial acetylation has been associated with reduced heart metabolism, elevated oxidative damage, and accelerated senescence [10]. Our data showed that the decreased level of the phosphorylated (active) form of AMPK (Thr172) in irradiated samples was associated with an increase in p-Akt (Ser473), which, however, was accompanied by a decrease in p-AMPK (Ser487) in our experiment. AMPK is a phosphoprotein with multiple catalytic subunits and different phosphorylation sites that regulate a wide range of cellular processes [45]. AMPK and Akt are in a complicated mutual antagonism to each other [46]. The two signalling pathways have often been shown to be inversely correlated, such that Akt negatively regulates AMPK phosphorylation [47,48]. It has been reported that transgenic mice overexpressing a constitutively active mutant of Akt or ageing-induced phosphorylation of Akt (Ser473) show reduced phosphorylation (Thr172) of cardiac AMPK [47,48]. Akt has been shown to phosphorylate AMPK at Ser487, resulting in reduced Thr172 phosphorylation [26]. However, it is known that AMPK also reversely inhibits Akt phosphorylation [46]. The knockdown of Akt did not affect AMPK activation in endothelial cells [49]. Furthermore, the study using the Akt-AMPK double knockout model showed that knockout of AMPK promotes cardiac ageing, an effect that is not attenuated by simultaneous knockout of Akt [50]. The detailed mechanisms contributing to the interaction between AMPK and Akt are not yet known, however, it is plausible that other upstream regulators are involved.

Akt/mTOR as part of the PI3K signalling pathway have been known to be linked to different cardiovascular pathology, including cardiac inflammation, fibrosis, hypertrophy, and atherosclerosis [51,52]. Activation of Akt/mTOR contributes to the ageing process by inhibiting autophagy [36,53] and regulating energy homeostasis and cellular senescence [54]. Chronic low dose rate radiation (4.1 mGy/h) has been shown to initiate premature senescence in Human Umbilical Vein Endothelial Cells (HUVECs) by the induction of the p53/p21 pathway [42]. The proteomic analysis indicated the role of the PI3K/Akt/mTOR pathway in this radiation-induced endothelial senescence. Supported by previous studies [10,55], we show increased mRNA levels of several senescence markers, including p21, p16, and CD44, in irradiated hearts, suggesting accumulation of senescent cells in heart tissue after chronic low dose irradiation. Indeed, such increased levels of p21 and p16 have been previously associated with an increased number of senescent cardiac cells in the aged heart and age-related heart diseases [56,57,58].

The proteomic data here revealed the downregulation of several proteins involved in cardiac metabolism in chronically irradiated hearts. In good agreement, the level of the phosphorylated (inactive form) of PPARα, a key regulator of cardiac lipid metabolism, increased following irradiation. The inactivation of the PPARα pathway and inhibition of lipid metabolism have been repeatedly reported in heart toxicity following high-dose radiation exposure [59,60,61]. Impairment in cardiac energy production was also observed in proteome profiles of the heart autopsies of the Mayak worker [28,29]. A decrease in fatty acid metabolism and energy production has been also reported in the ageing heart in association with lower expression of cardiac PPARα [62,63].

A balance between AMPK and mTOR signalling is important to sustain PPARα activity in the heart [64,65]. SIRT1 has been shown to interact with PPARα directly and facilitates PGC-1/PPARα interactions [66]. SIRT3 and SIRT1 deacetylate PGC-1 and thereby activate as a cofactor in PPAR signalling [67,68]. We have shown decreased SIRT1 and SIRT3 activity associated with changes in the acetylation state of cardiac mitochondrial proteins in chronically irradiated ApoE^−/−^ mice [10].

Proteomics data here indicated a cluster of altered proteins belonging to oxidative stress response, particularly in glutathione metabolism. Persistently imbalanced reactive oxygen species (ROS) homeostasis level has been shown in cardiac tissue following irradiation [69,70]. Our analysis confirmed significantly decreased activities of antioxidant proteins that coincided with an imbalance of GSS/GSH resulting in enhanced protein glutathionylation and lipid peroxidation as markers of oxidative damage. Radiation-associated increase of the oxidized and glutathionylated proteins suggests the accumulation of dysfunctional proteins following irradiation. Stress-induced modifications of proteins have also been reported in mouse and human heart tissues following chronic irradiation [10,28]. Such modifications lead to the inactivation and degradation of proteins [71,72]. Cardiac ageing is also known to be associated with an imbalance between the production of ROS and a decrease in the cellular antioxidant defences, leading to increased inflammation and oxidative damage [73,74].

Several collagens were upregulated here in the proteomics and transcriptomics data and predicted to be regulated by activated TGFβ. Cardiac fibrosis as an excessive deposition of ECM is a known consequence of ionizing radiation found in patients who received radiotherapy [1,75]. We have previously shown an increase in proteins involved in the ECM remodelling after high doses of local heart irradiation, with the activation of TGFβ playing a central role [60]. The increased concentration of TGFβ has been reported in the blood serum of Mayak workers chronically exposed to low doses of external gamma rays, compared to the control [76]. In the same line, excessive deposition of collagen was observed in FFPE cardiac autopsies of Mayak workers compared to controls [11]. Activation of fibrogenic signalling and alteration in the production and turnover of ECM proteins are known processes involved in cardiac fibrosis in the ageing heart [77,78]. Such imbalance in ECM homeostasis causes the accumulation of damaged or abnormal proteins in the heart and results in cardiac dysfunction [77,78].

**Figure 8:**
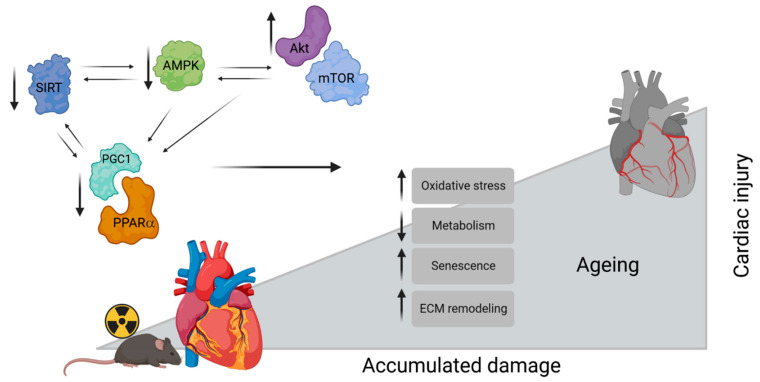
A proposed model for the role of the main ageing-associated signalling pathways in cardiac injury after chronic irradiation. Created with BioRender.com.

Our data highlight one potential mechanism for the development of cardiac effects of chronic irradiation and the similarity of the signalling pathways affected to those contributing to premature cardiac ageing. A putative interaction between the irradiation and the main ageing-associated signalling pathways in cardiac injury after chronic irradiation is shown in a model in Figure 8. The model proposes an interplay between the observed alterations in the SIRT, AMPK, and mTOR signalling pathways. Chronic radiation deactivates the SIRT pathway and activates the mTOR pathway in the heart. The AMPK pathway plays a critical role in the interconnectivity between the two molecular events. Both SIRT and mTOR pathways regulate the PPARα to maintain cardiac metabolism and inflammatory homeostasis. Alterations in these signalling pathways are proposed to contribute to impaired cardiac metabolism, increased oxidative damage, ECM remodelling, accelerated senescence, and accumulated damage (Figure 8).

Although more research is needed to elucidate the mechanistic link between chronic low dose radiation exposure and CVD, these data support the hypothesis that ionizing radiation can accelerate cardiac ageing and thereby result in increased disease risk. Further studies are still required to evaluate the impact of various modifiable and nonmodifiable factors (such as age, gender, and lifestyle) on CVD induced by chronic low dose irradiation. It remains to identify the cardiac cells that are key players in radiation-induced ageing and to clarify the contribution of cell communication to this process. Bearing in mind that disruption of cardiovascular metabolism seems to play a crucial role in CVD after chronic irradiation [10,11,43], a possible approach would be to evaluate the effect of pharmacological activation of PPARα, a key transcription factor of metabolism in the prevention of radiation-associated heart disease [79].

## 5. Conclusions

Data presented here collectively showed that low dose chronic total body irradiation induced biological alterations in mouse cardiac tissues that are similar to the effects of premature cardiac ageing. The study found a series of alterations in ageing-associated heart pathology contributing to cardiac injury as reported in occupationally exposed populations. A deeper understanding of the molecular mechanisms of CVD following chronic low-dose irradiation will allow for the identification of target molecules or pathways that can be used in bioassays to measure endpoints relevant to radiation risk assessment [80].

## Data Availability

Data have been deposited into the RBstore public database: https://www.storedb.org/store_v3/study.jsp?studyId=1106 (accessed on 1 February 2023).

## Supplementary Materials

The following supporting information can be downloaded at: https://www.mdpi.com/article/10.3390/cancers15133417/s1, Figure S1: Masson trichrome staining for evaluation of collagen deposition in the right ventricle, Figure S2: Immunoblot analysis of AMPK, p-AMPK (T172) and Akt and p-Akt (S473). And Original Blots; Table S1: All identified and quantified proteins; Table S2: Significantly deregulated proteins in the heart after chronic exposure to an accumulated dose of 0.3 Gy; Table S3: Significantly deregulated proteins in the heart after chronic exposure to an accumulated dose of 6 Gy; Table S4: Affected pathways in the heart after chronic exposure to an accumulated dose of 0.3 Gy; Table S5: Affected pathways in the heart after chronic exposure to an accumulated dose of 6 Gy; Table S6: Main affected pathways, upstream regulators and associated cardiac toxicity in irradiated heart; Table S7: All senescence-related mRNA (Ct Values) in control and chronically irradiated hearts; Table S8: Significantly deregulated senescence-related mRNA in the heart after chronic exposure to an accumulated dose of 0.3 Gy; Table S9: Significantly deregulated senescence-related mRNA in the heart after chronic exposure to an accumulated dose of 6 Gy.
