# Supplementary material for: Late Effects of Chronic Low Dose Rate Total Body Irradiation on the Heart Proteome of ApoE−/− Mice Resemble Premature Cardiac Ageing
Source: Cancers (Basel). 2023 Jun 29;15(13):3417. doi: 10.3390/cancers15133417 (PMC10340334; doi:10.3390/cancers15133417)
Supplement: Supplementary file 1 [file cancers-15-03417-s001.zip › OriginalBlots20062023.pptx]

## Slide 1
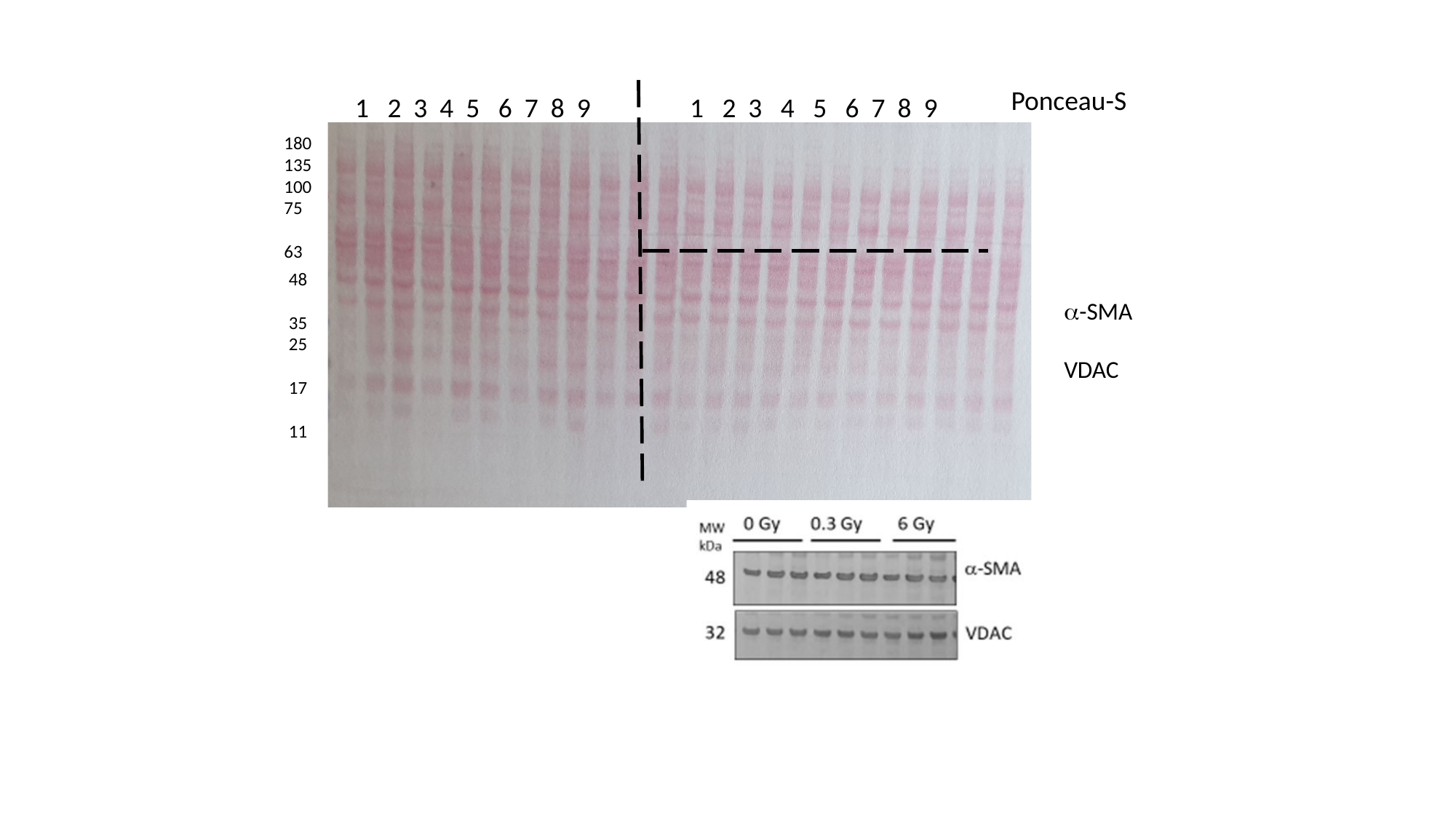

Ponceau-S
1 2 3 4 5 6 7 8 9
1 2 3 4 5 6 7 8 9
180
135
100
75
63
48
35
25
17
11
 a-SMA
 VDAC

## Slide 2
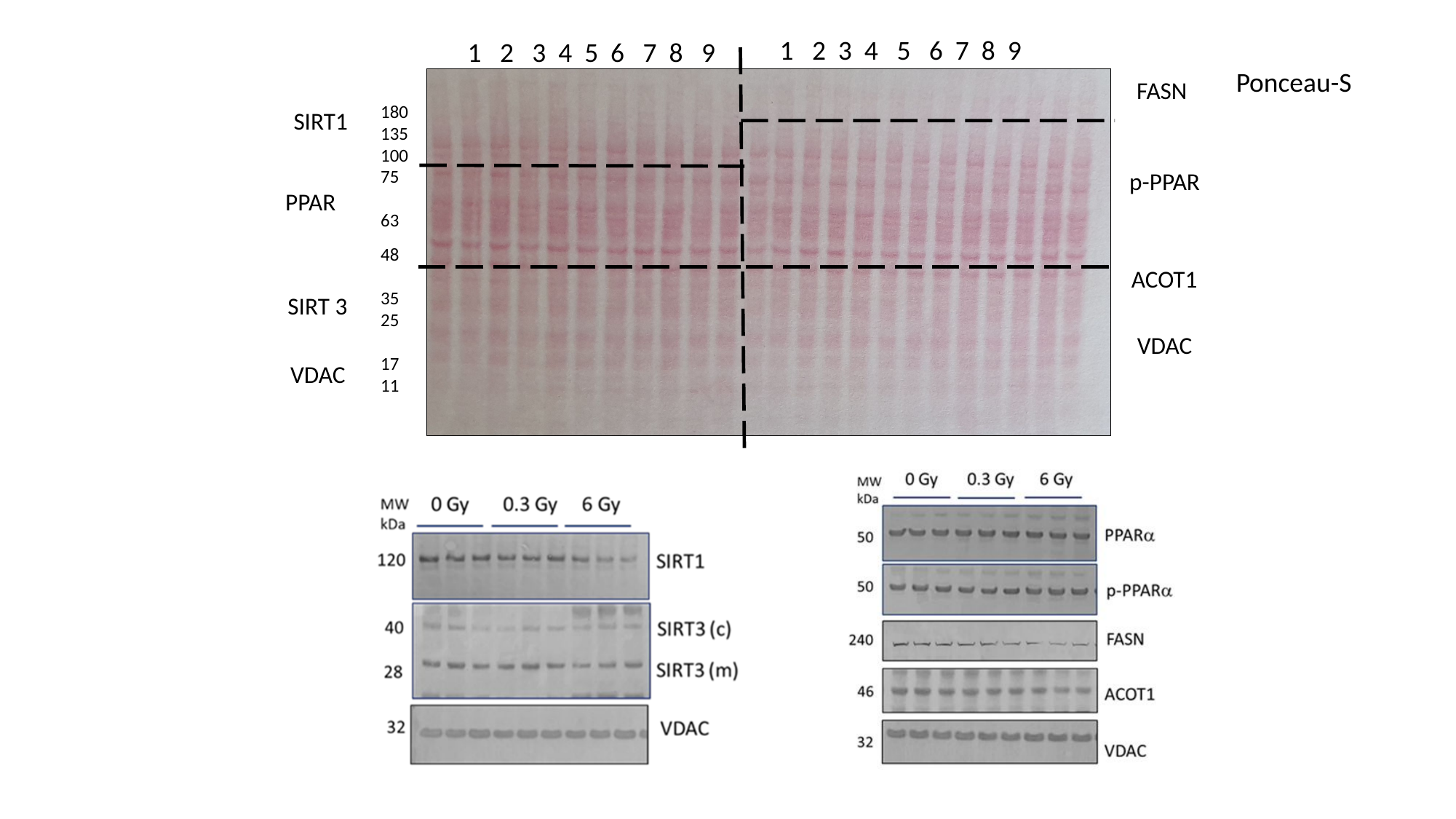

1 2 3 4 5 6 7 8 9
1 2 3 4 5 6 7 8 9
Ponceau-S
FASN
180
135
100
75
63
48
35
25
17
11
SIRT1
 p-PPAR
PPAR
ACOT1
SIRT 3
VDAC
VDAC

## Slide 3
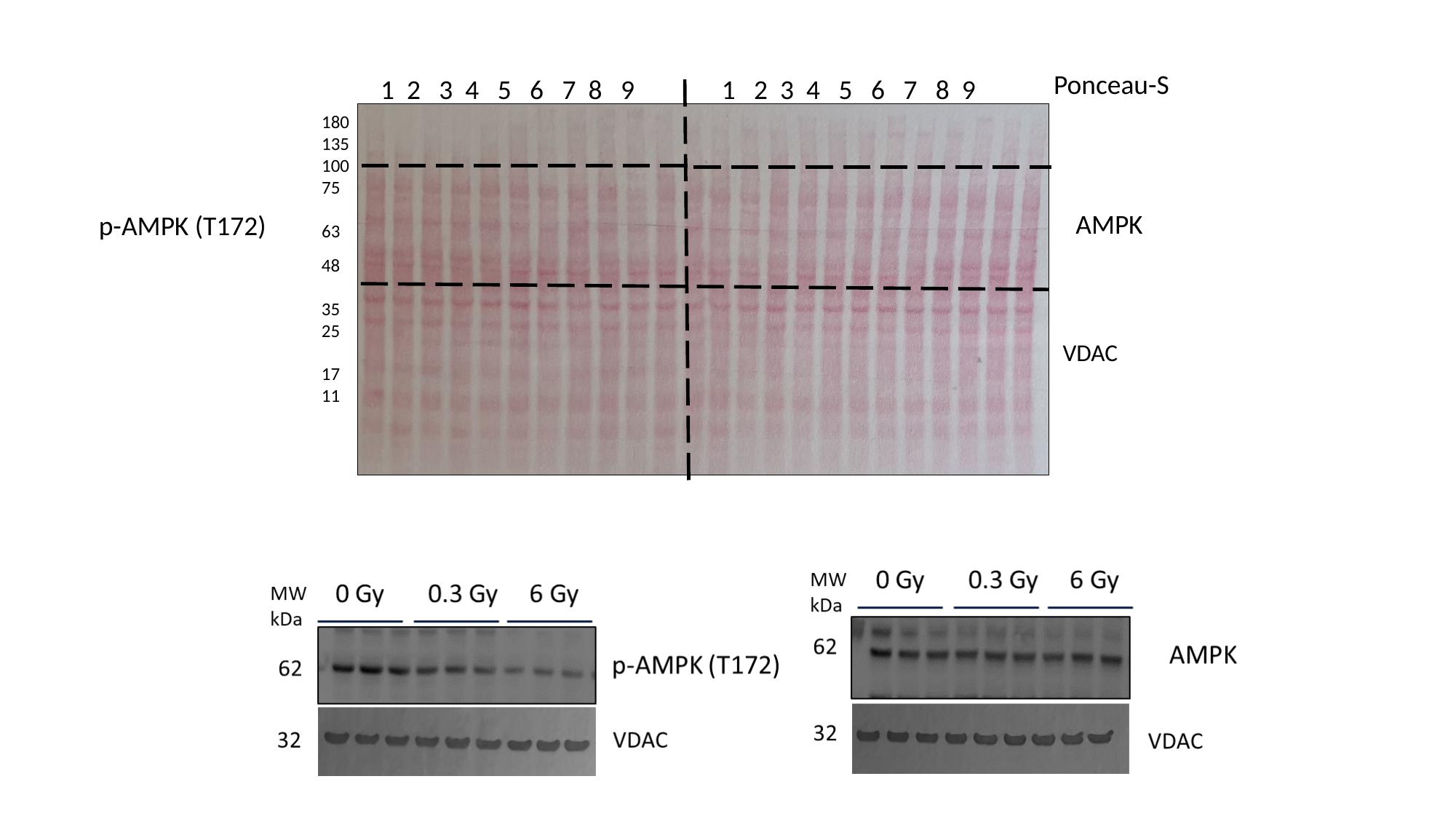

Ponceau-S
1 2 3 4 5 6 7 8 9
1 2 3 4 5 6 7 8 9
180
135
100
75
63
48
35
25
17
11
AMPK
 p-AMPK (T172)
VDAC

## Slide 4
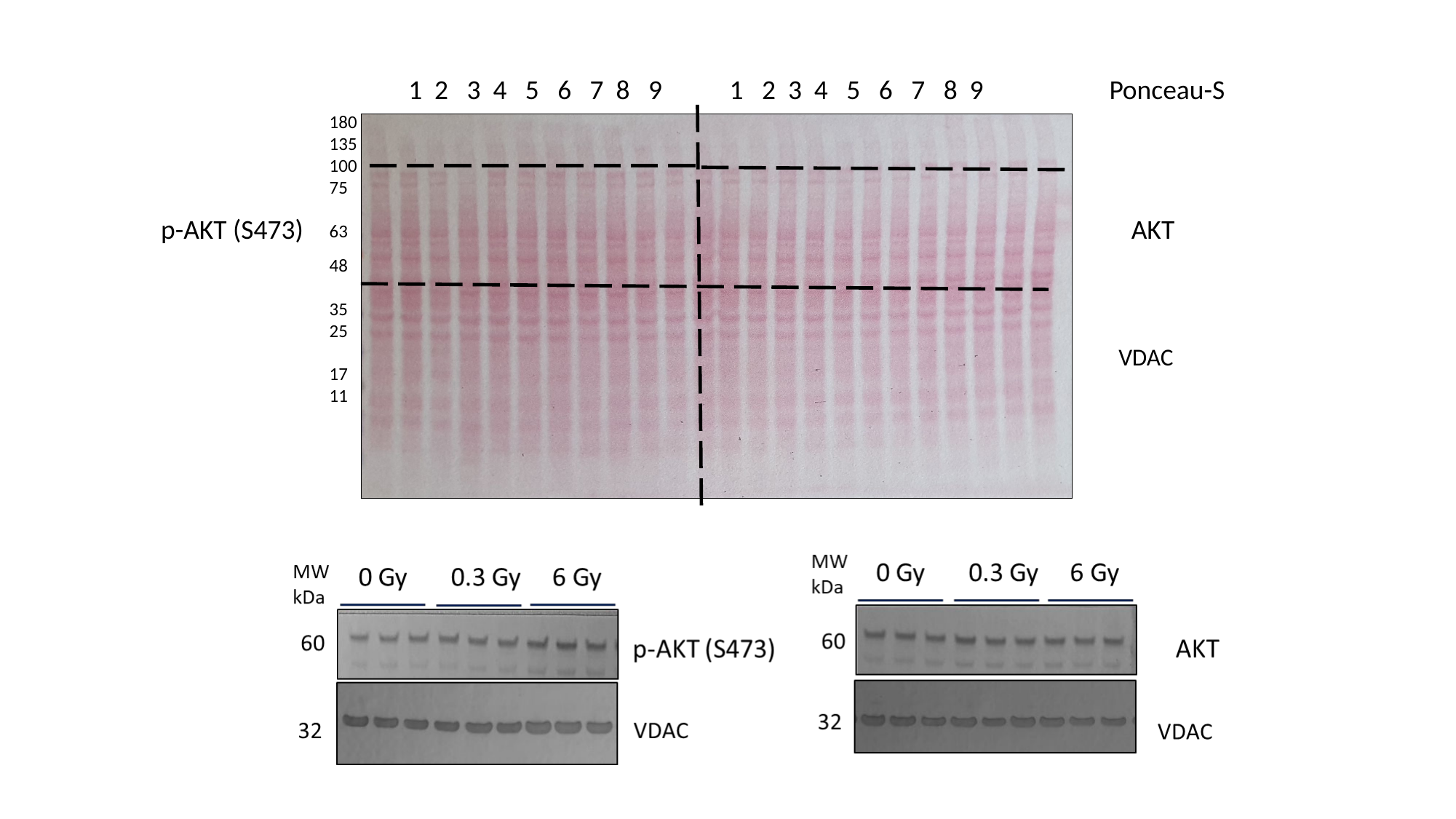

1 2 3 4 5 6 7 8 9
1 2 3 4 5 6 7 8 9
180
135
100
75
63
48
35
25
17
11
Ponceau-S
 p-AKT (S473)
AKT
VDAC

## Slide 5
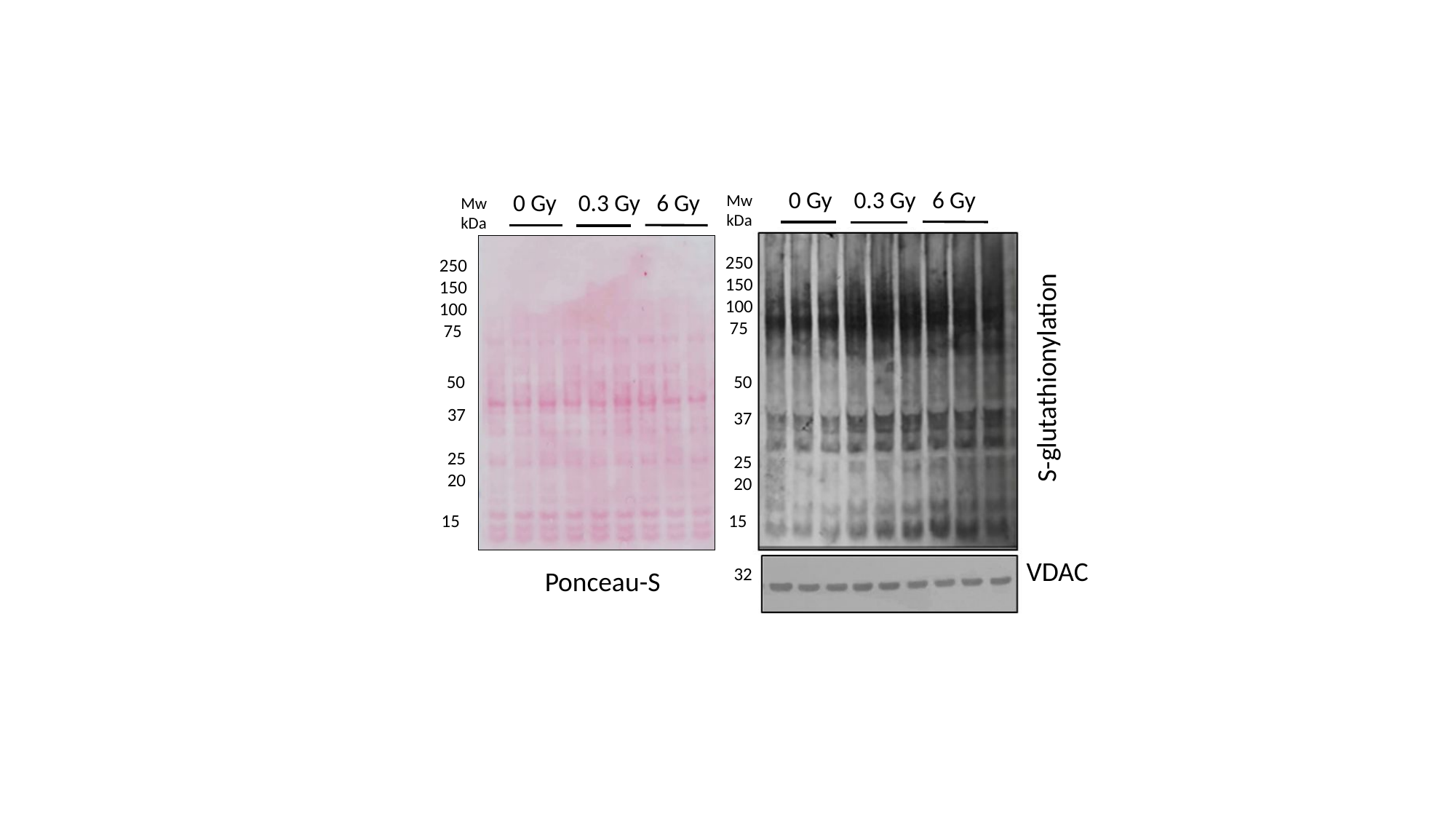

0 Gy 0.3 Gy 6 Gy
Mw
kDa
32
 0 Gy 0.3 Gy 6 Gy
Mw
kDa
250
150
100
 75
50
37
25
20
15
250
150
100
 75
S-glutathionylation
50
37
25
20
15
VDAC
Ponceau-S
