# Supplementary material for: Late Effects of Chronic Low Dose Rate Total Body Irradiation on the Heart Proteome of ApoE−/− Mice Resemble Premature Cardiac Ageing
Source: Cancers (Basel). 2023 Jun 29;15(13):3417. doi: 10.3390/cancers15133417 (PMC10340334; doi:10.3390/cancers15133417)
Supplement: Supplementary file 1 [file cancers-15-03417-s001.zip › Supplementary Figures_20062023.pptx]

## Slide 1
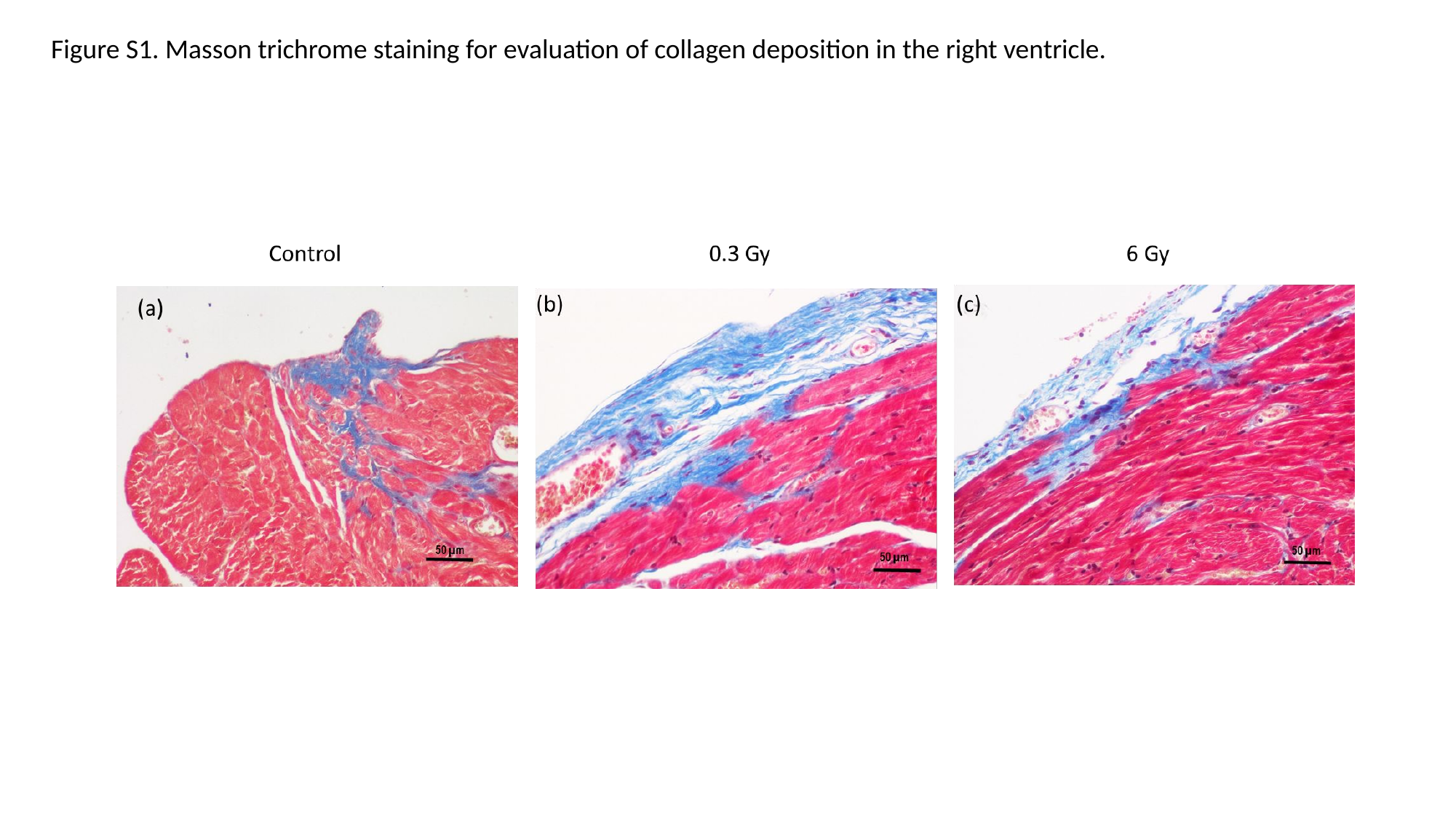

Figure S1. Masson trichrome staining for evaluation of collagen deposition in the right ventricle.

## Slide 2
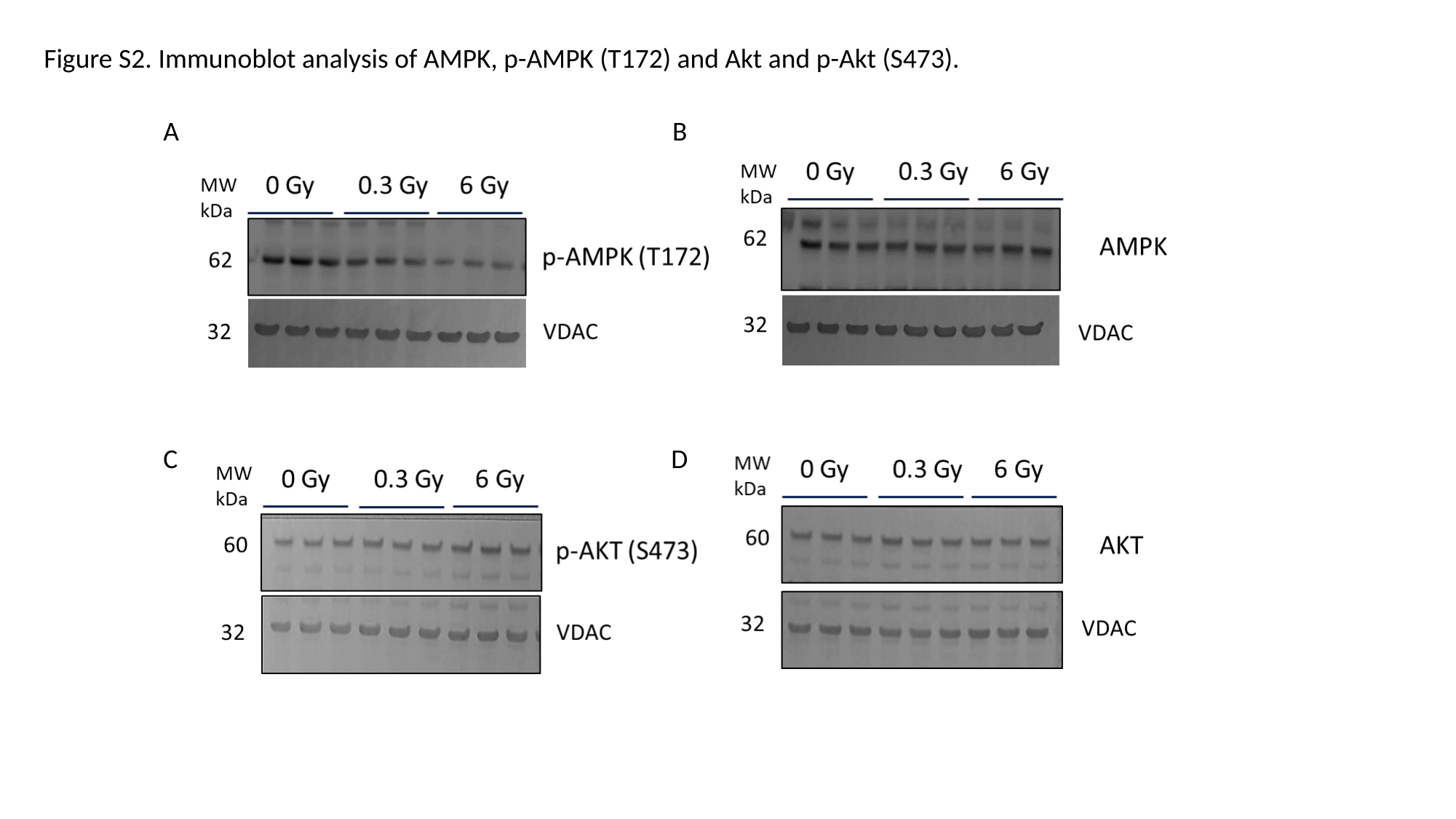

Figure S2. Immunoblot analysis of AMPK, p-AMPK (T172) and Akt and p-Akt (S473).
A B
C D
